# Supplementary material for: Neural crest-specific deletion of Rbfox2 in mice leads to craniofacial abnormalities including cleft palate
Source: eLife. 2019 Jun 26;8:e45418. doi: 10.7554/eLife.45418 (PMC6663295; doi:10.7554/eLife.45418)
Supplement: Figure 5—source data 1. [file elife-45418-fig5-data1.docx]

**Figure 5- Source data 1.** A complete list of transcripts identified by MISO analysis.

| **Event_Nam** | **Transcript_ID** | **Gene** | **Difference (KO/Cont.)** |
| --- | --- | --- | --- |
| ENSMUSG00000026193.15 | ENSMUST00000188894.6 | Fn1 | 33.33 |
| ENSMUSG00000023008.18 | ENSMUST00000120633.1 | Fmnl3 | 29.80 |
| ENSMUSG00000030669.13 | ENSMUST00000032907.8 | Calca | 6.82 |
| ENSMUSG00000027750.16 | ENSMUST00000117373.7 | Postn | 3.41 |
| ENSMUSG00000028284.13 | ENSMUST00000037607.10 | Map3k7 | 3.00 |
| ENSMUSG00000023994.13 | ENSMUST00000162460.7 | Nfya | 2.92 |
| ENSMUSG00000026670.15 | ENSMUST00000111350.9 | Uap1 | 2.62 |
| ENSMUSG00000050050.17 | ENSMUST00000150359.1 | Ccdc158 | 2.56 |
| ENSMUSG00000021779.16 | ENSMUST00000022303.13 | Thrb | 2.54 |
| ENSMUSG00000032374.14 | ENSMUST00000070522.13 | Plod2 | 2.48 |
| ENSMUSG00000064105.12 | ENSMUST00000077666.4 | Cnnm2 | 2.41 |
| ENSMUSG00000032417.10 | ENSMUST00000034988.9 | Rwdd2a | 2.40 |
| ENSMUSG00000032492.14 | ENSMUST00000198865.4 | Pth1r | 2.38 |
| ENSMUSG00000086894.2 | ENSMUST00000156467.1 | Gm15708 | 2.38 |
| ENSMUSG00000031328.15 | ENSMUST00000101454.8 | Flna | 2.27 |
| ENSMUSG00000028550.15 | ENSMUST00000152121.1 | Atg4c | 2.24 |
| ENSMUSG00000026896.14 | ENSMUST00000112459.3 | Ifih1 | 2.16 |
| ENSMUSG00000005057.13 | ENSMUST00000196397.4 | Sh2b2 | 2.15 |
| ENSMUSG00000036292.13 | ENSMUST00000036174.9 | Gramd1c | 2.01 |
| ENSMUSG00000035172.15 | ENSMUST00000164474.7 | Plekhh3 | 1.97 |
| ENSMUSG00000031170.14 | ENSMUST00000033512.10 | Slc38a5 | 1.96 |
| ENSMUSG00000028294.15 | ENSMUST00000029971.11 | Cfap206 | 1.95 |
| ENSMUSG00000070336.3 | ENSMUST00000093939.3 | Fbxo47 | 1.93 |
| ENSMUSG00000030745.9 | ENSMUST00000033000.7 | Il21r | 1.82 |
| ENSMUSG00000044542.3 | ENSMUST00000051159.2 | Prop1 | 1.76 |
| ENSMUSG00000022139.16 | ENSMUST00000088419.12 | Mbnl2 | 1.70 |
| ENSMUSG00000101144.2 | ENSMUST00000187589.1 | Gm29054 | 1.70 |
| ENSMUSG00000052387.15 | ENSMUST00000099566.3 | Trpm3 | 1.60 |
| ENSMUSG00000034764.15 | ENSMUST00000041178.11 | 1700006J14Rik | 1.60 |
| ENSMUSG00000015647.9 | ENSMUST00000015791.5 | Lama5 | 1.56 |
| ENSMUSG00000050675.7 | ENSMUST00000055184.6 | Gp1ba | 1.56 |
| ENSMUSG00000025893.8 | ENSMUST00000212221.1 | Kbtbd3 | 1.54 |
| ENSMUSG00000052056.14 | ENSMUST00000109155.7 | Zfp217 | 1.49 |
| ENSMUSG00000024044.16 | ENSMUST00000080208.6 | Epb41l3 | 1.48 |
| ENSMUSG00000030882.18 | ENSMUST00000145988.8 | Dnhd1 | 1.48 |
| ENSMUSG00000079056.12 | ENSMUST00000103215.10 | Kcnip3 | 1.47 |
| ENSMUSG00000031644.19 | ENSMUST00000034065.13 | Nek1 | 1.44 |
| ENSMUSG00000028175.15 | ENSMUST00000120272.7 | Depdc1a | 1.31 |
| ENSMUSG00000033342.13 | ENSMUST00000106473.4 | Plppr5 | 1.26 |
| ENSMUSG00000001348.14 | ENSMUST00000165735.7 | Acp5 | 1.24 |
| ENSMUSG00000022372.14 | ENSMUST00000100572.9 | Sla | 1.00 |
| ENSMUSG00000052056.14 | ENSMUST00000063710.12 | Zfp217 | 0.71 |
| ENSMUSG00000028435.8 | ENSMUST00000055327.7 | Aqp3 | 0.68 |
| ENSMUSG00000020334.6 | ENSMUST00000020586.6 | Slc22a4 | 0.67 |
| ENSMUSG00000085715.2 | ENSMUST00000152916.1 | Tsix | 0.65 |
| ENSMUSG00000025026.14 | ENSMUST00000050096.13 | Add3 | 0.65 |
| ENSMUSG00000032624.15 | ENSMUST00000096766.10 | Eml4 | 0.64 |
| ENSMUSG00000034706.16 | ENSMUST00000069325.13 | Dnaic2 | 0.63 |
| ENSMUSG00000056296.16 | ENSMUST00000070323.11 | Synpr | 0.62 |
| ENSMUSG00000025893.8 | ENSMUST00000049648.8 | Kbtbd3 | 0.62 |
| ENSMUSG00000020227.10 | ENSMUST00000020448.10 | Irak3 | 0.60 |
| ENSMUSG00000021779.16 | ENSMUST00000091471.10 | Thrb | 0.60 |
| ENSMUSG00000035172.15 | ENSMUST00000043397.13 | Plekhh3 | 0.59 |
| ENSMUSG00000038072.14 | ENSMUST00000114952.7 | Galnt11 | 0.59 |
| ENSMUSG00000040543.16 | ENSMUST00000075258.12 | Pitpnm3 | 0.59 |
| ENSMUSG00000044566.15 | ENSMUST00000089840.4 | Cage1 | 0.58 |
| ENSMUSG00000036292.13 | ENSMUST00000179565.7 | Gramd1c | 0.58 |
| ENSMUSG00000064105.12 | ENSMUST00000099373.10 | Cnnm2 | 0.55 |
| ENSMUSG00000061132.13 | ENSMUST00000054769.6 | Blnk | 0.53 |
| ENSMUSG00000022139.16 | ENSMUST00000167459.1 | Mbnl2 | 0.51 |
| ENSMUSG00000052384.13 | ENSMUST00000099991.10 | Nrros | 0.49 |
| ENSMUSG00000101144.2 | ENSMUST00000194148.1 | Gm29054 | 0.48 |
| ENSMUSG00000047040.9 | ENSMUST00000054311.5 | Prr15l | 0.48 |
| ENSMUSG00000067006.12 | ENSMUST00000086701.12 | Serpinb5 | 0.46 |
| ENSMUSG00000023008.18 | ENSMUST00000088233.12 | Fmnl3 | 0.46 |
| ENSMUSG00000026896.14 | ENSMUST00000028259.11 | Ifih1 | 0.46 |
| ENSMUSG00000024044.16 | ENSMUST00000112680.7 | Epb41l3 | 0.44 |
| ENSMUSG00000070336.3 | ENSMUST00000125403.1 | Fbxo47 | 0.44 |
| ENSMUSG00000086894.2 | ENSMUST00000198873.1 | Gm15708 | 0.44 |
| ENSMUSG00000005057.13 | ENSMUST00000005188.13 | Sh2b2 | 0.41 |
| ENSMUSG00000032492.14 | ENSMUST00000166716.7 | Pth1r | 0.40 |
| ENSMUSG00000050675.7 | ENSMUST00000108551.2 | Gp1ba | 0.36 |
| ENSMUSG00000026193.15 | ENSMUST00000055226.12 | Fn1 | 0.35 |
| ENSMUSG00000032374.14 | ENSMUST00000160359.1 | Plod2 | 0.35 |
| ENSMUSG00000050050.17 | ENSMUST00000151180.7 | Ccdc158 | 0.33 |
| ENSMUSG00000028284.13 | ENSMUST00000080933.12 | Map3k7 | 0.32 |
| ENSMUSG00000028175.15 | ENSMUST00000106041.2 | Depdc1a | 0.29 |
| ENSMUSG00000032417.10 | ENSMUST00000179212.2 | Rwdd2a | 0.28 |
| ENSMUSG00000029207.16 | ENSMUST00000160870.7 | Apbb2 | 0.27 |
| ENSMUSG00000079056.12 | ENSMUST00000028850.14 | Kcnip3 | 0.26 |
| ENSMUSG00000029086.15 | ENSMUST00000087441.10 | Prom1 | 0.16 |
