# Supplementary material for: Neural crest-specific deletion of Rbfox2 in mice leads to craniofacial abnormalities including cleft palate
Source: eLife. 2019 Jun 26;8:e45418. doi: 10.7554/eLife.45418 (PMC6663295; doi:10.7554/eLife.45418)
Supplement: Figure 5—source data 2. [file elife-45418-fig5-data2.docx]

**Figure 5- Source data 2.** A complete list of transcripts identified by Cuffdiff analysis.

| **Gene_ID** | **Transcript_ID** | **Gene** | **Fold change (KO/Cont.)** | **p_value** |
| --- | --- | --- | --- | --- |
| ENSMUSG00000058835.14 | ENSMUST00000149719.7 | Abi1 | 4.26 | 0.0007 |
| ENSMUSG00000027750.16 | ENSMUST00000117373.7 | Postn | 3.49 | 0.00005 |
| ENSMUSG00000021709.14 | ENSMUST00000191275.6 | Erbb2ip | 3.48 | 0.00055 |
| ENSMUSG00000025290.16 | ENSMUST00000169826.1 | Rps24 | 3.42 | 0.0274 |
| ENSMUSG00000030704.14 | ENSMUST00000098252.4 | Rab6a | 2.76 | 0.0382 |
| ENSMUSG00000028284.13 | ENSMUST00000037607.10 | Map3k7 | 2.73 | 0.00015 |
| ENSMUSG00000026670.15 | ENSMUST00000111350.9 | Uap1 | 2.33 | 0.0008 |
| ENSMUSG00000023994.13 | ENSMUST00000162460.7 | Nfya | 2.29 | 0.0018 |
| ENSMUSG00000032374.14 | ENSMUST00000070522.13 | Plod2 | 2.23 | 0.00005 |
| ENSMUSG00000036435.13 | ENSMUST00000087133.10 | Exoc1 | 2.18 | 0.00115 |
| ENSMUSG00000021268.17 | ENSMUST00000129245.7 | Meg3 | 0.50 | 0.0015 |
| ENSMUSG00000031328.15 | ENSMUST00000033699.12 | Flna | 0.50 | 0.00005 |
| ENSMUSG00000029068.16 | ENSMUST00000139066.7 | Ccnl2 | 0.47 | 0.0014 |
| ENSMUSG00000024921.16 | ENSMUST00000025862.13 | Smarca2 | 0.47 | 0.0004 |
| ENSMUSG00000023008.18 | ENSMUST00000088233.12 | Fmnl3 | 0.45 | 0.00005 |
| ENSMUSG00000039953.13 | ENSMUST00000039144.6 | Clstn1 | 0.44 | 0.0223 |
| ENSMUSG00000023994.13 | ENSMUST00000078800.12 | Nfya | 0.43 | 0.00145 |
| ENSMUSG00000038074.16 | ENSMUST00000046520.12 | Fkbp14 | 0.42 | 0.0001 |
| ENSMUSG00000022139.16 | ENSMUST00000167459.1 | Mbnl2 | 0.42 | 0.00005 |
| ENSMUSG00000027750.16 | ENSMUST00000107985.9 | Postn | 0.41 | 0.00005 |
| ENSMUSG00000031078.15 | ENSMUST00000103079.3 | Cttn | 0.40 | 0.00005 |
| ENSMUSG00000027750.16 | ENSMUST00000073012.12 | Postn | 0.39 | 0.00005 |
| ENSMUSG00000002732.14 | ENSMUST00000176815.1 | Fkbp7 | 0.38 | 0.00145 |
| ENSMUSG00000026670.15 | ENSMUST00000111351.9 | Uap1 | 0.38 | 0.00005 |
| ENSMUSG00000032366.15 | ENSMUST00000113707.8 | Tpm1 | 0.37 | 0.00005 |
| ENSMUSG00000036435.13 | ENSMUST00000049469.12 | Exoc1 | 0.37 | 0.00005 |
| ENSMUSG00000002732.14 | ENSMUST00000002809.13 | Fkbp7 | 0.34 | 0.00005 |
| ENSMUSG00000028284.13 | ENSMUST00000080933.12 | Map3k7 | 0.33 | 0.00005 |
| ENSMUSG00000032374.14 | ENSMUST00000160359.1 | Plod2 | 0.33 | 0.00005 |
| ENSMUSG00000026193.15 | ENSMUST00000055226.12 | Fn1 | 0.26 | 0.00005 |
| ENSMUSG00000032366.15 | ENSMUST00000113685.9 | Tpm1 | 0.24 | 0.01175 |
| ENSMUSG00000029086.15 | ENSMUST00000087441.10 | Prom1 | 0.24 | 0.00455 |
| ENSMUSG00000086503.3 | ENSMUST00000127786.2 | Xist | 0.18 | 0.00005 |
